# Mortality from Congenital Heart Disease in Mexico: A Problem on the Rise

**DOI:** 10.1371/journal.pone.0150422

**Published:** 2016-03-03

**Authors:** José Luis Torres-Cosme, Constanza Rolón-Porras, Mónica Aguinaga-Ríos, Pedro Manuel Acosta-Granado, Enrique Reyes-Muñoz, Teresa Murguía-Peniche

**Affiliations:** 1 Research Division, Community Interventions, Instituto Nacional de Perinatología ‘Isidro Espinosa de los Reyes’, Mexico City, Mexico; 2 Genetics Department, Research Division, Instituto Nacional de Perinatología ‘Isidro Espinosa de los Reyes’, Mexico City, Mexico; 3 Neurosciences Department, Instituto Nacional de Perinatología ‘Isidro Espinosa de los Reyes’, Mexico City, Mexico; 4 Endocrinology Department, Instituto Nacional de Perinatología ‘Isidro Espinosa de los Reyes’, Mexico City, Mexico; 5 Faculty of Health Sciences, Universidad Panamericana, Mexico City, Mexico; University of Barcelona, SPAIN

## Abstract

**Background and Objectives:**

Temporal trends in mortality from congenital heart disease (CHD) vary among regions. It is therefore necessary to study this problem in each country. In Mexico, congenital anomalies were responsible for 24% of infant mortality in 2013 and CHD represented 55% of total deaths from congenital anomalies among children under 1 year of age. The objectives of this study were to analyze the trends in infant mortality from CHD in Mexico (1998 to 2013), its specific causes, age at death and associated socio-demographic factors.

**Methods:**

Population-based study which calculated the compounded annual growth rate of death rom CHD between 1998 and 2013. Specific causes, age at which death from CHD occurred and risk factors associated with mortality were analyzed for the year 2013.

**Results:**

Infant mortality from CHD increased 24.8% from 1998 to 2013 (114.4 to 146.4/ 100,000 live births). A total of 3,593 CHD deaths occurred in 2013; the main causes were CHD with left-to-right shunt (n = 487; 19.8/100,000 live births) and cyanotic heart disease (n = 410; 16.7/100,000). A total of 1,049 (29.2%) deaths from CHD occurred during the first week of life. Risk factors associated with mortality from CHD were, in order of magnitude: non-institutional birth, rural area, birth in a public hospital and male sex.

**Conclusions:**

Mortality from CHD has increased in Mexico. The main causes were CHD with left-to-right shunt, which are not necessarily fatal if treated promptly. Populations vulnerable to death from CHD were identified. Approximately one-third of the CHD occurred during the first week of life. It is important to promote early diagnosis, especially for non-institutional births.

## Introduction

Worldwide, mortality of children under 5 years of age decreased 47% from 1990 to 2015, from 90.6 to 42.5 deaths per 1,000 live births [1]. This was achieved primarily by decreasing diarrheal and respiratory infections as well as diseases that can be prevented by vaccinations. Meanwhile, congenital anomalies have become a more significant cause of death among children under 5 years of age, increasing from 5% of deaths in 2000 to 7% in 2013; and this increase was even larger in the Americas, from 15 to 21% during this same period [2]. Furthermore, congenital heart disease accounts for nearly one-third of congenital anomalies [3].

Temporal trends in congenital heart disease (CHD) vary among countries. Mortality from CHD decreased over recent years in Canada and in 16 European countries, particularly for children under 1 year of age [4–6]. A decrease in mortality from CHD was also observed in the United States (U.S.) between 1970 and 1997, contributing to a 59% reduction in infant mortality during this period [7]. In contrast, the overall mortality rate from CHD in China increased 62% between 2003 and 2010 [8].

Mortality from CHD has increased in some countries, which may actually reflect a higher number of CHD patients diagnosed and registered at birth and higher necropsy rates for perinatal deaths, rather than an increase in the prevalence of the disease. This deserves further research.

In Mexico, significant efforts have been undertaken over recent years to decrease infant mortality among children under 1 year of age. Thus, between 1990 and 2010 the infant mortality rate decreased from 24.0 to 11.8 per 1000 live births [9]. Nevertheless, mortality from CHD was found to increase in both absolute and relative terms. In the year 2000, CHD caused the death of 2,596 children under 1 year of age, representing 6.7% of total deaths in this age group. By 2008, mortality from CHD increased to 2,848, representing 9.6% of the total [10].

Current and detailed information is required to identify national mortality trends related to all CHD in the infant population, and needs to be based on the ICD-10 in order to make international comparisons. Information is also needed that is useful to those responsible for making decisions about children’s public health in Mexico as well as to international health agencies, about a problem which seems to be on the rise in developing nations and which is a global health concern.

The main objective of the present study is to analyze trends in mortality from CHD in Mexico between 1998 and 2013. Secondary objectives are identifying the principal causes of death from CHD, determining the age at death from CHD and analyzing socio-demographic factors associated with death from CHD.

## Methods

### Study period and variables included

This is a population-based study conducted in Mexico which analyzes trends in infant mortality from CHD from 1998—the year when this country began to use the 10th revision of the ICD (ICD-10) [11]—until 2013. This made it possible to obtain a more detailed and specific description of CHD. For the purpose of international comparisons, all deaths from CHD corresponding to ICD-10 codes Q20-Q28 were included [12]. The study also included the following variables contained in the death certificates: sex, age, primary place of residence, state, size of locality and place where the death occurred. These data did not allow for estimating the proportion of preterm patients or determining diagnoses associated with chromosomal abnormalities. Fetal deaths were not included in the analysis because Mexico does not have a national surveillance program to collect all diagnoses of fetal cardiac disease.

The number of surgeons, cardiologists and pediatricians registered in Mexico in 2003 and 2012 were included in the analysis; these are the years with known information and were used for comparison purposes.

### Data sources

The information was obtained from all death certificates. These are routinely coded by the General Department of Health Information (DGIS, Spanish acronym) which produces electronic databases available to the public through the National System for Health Information (SINAIS, Spanish acronym), at the DGIS webpage [10]. The data for number of live registered births (LB) and socio-demographic information were obtained from the webpage provided by the National Institute of Statistics and Geography (INEGI, Spanish acronym) [13].

The number of surgeons, cardiologists and pediatricians registered in Mexico corresponds to the job positions reported by public hospitals and the Social Security Institute, according to the information from the DGIS [14]. This information was used to calculate rates of specialists per 1,000 LB.

### Operational definitions

Rates were calculated based on the number of deaths from CHD analyzed by this study for each specific year divided by the number of LB for each corresponding year, multiplied by 100,000.

The Functional Classification of CHD proposed by Reller et al [15] was adapted for this specific analysis, which divides heart defects into four subgroups: left-to-right shunt, cyanotic heart disease, obstructive defects of the left heart and obstructive defects of the right heart. All cases of patent ductus arteriosus were included since gestational age was not available.

The classification proposed by the 2012 Mexican National Health and Nutrition Survey [16] was used to define the regional geographic distribution of the 32 states in the country, as follows: North (Baja California, Baja California Sur, Coahuila, Chihuahua, Durango, Nuevo León, Sinaloa, Sonora, Tamaulipas and Zacatecas); Central (Aguascalientes, Colima, Guanajuato, Hidalgo, Jalisco, Mexico State, Michoacán, Nayarit, Querétaro, San Luis Potosí and Tlaxcala); Mexico City (Federal District); and South (Campeche, Chiapas, Guerrero, Morelos, Oaxaca, Puebla, Quintana Roo, Tabasco, Veracruz and Yucatán). Rural areas were defined as residential localities with 2,500 or less inhabitants and urban areas as regions with a larger population [9].

### Statistical analysis

1. The trend in CHD mortality rates per 100,000 LB was determined for the years 1998 to 2013. The CHD mortality rate and the compounded annual rate of growth (CARG) were calculated to determine the accumulated adjusted change in the rate for the period 1998–2013, according to the following formula: [final value/beginning value]^[1/number of years]^
2. In 2013, the total deaths from CHD that occurred during the first year of life were calculated by postnatal period, classified by our data source as follows: first 24 hours of life, 1 to 6 days of life, 7 to 27 days of life and 28 days to 11 months of life. The number of deaths from CHD per day was estimated for each period.
3. For the year 2013, a univariate analysis was performed [(odds ratio (OR); 95% confidence interval (95%CI)] and the Mantel-Haenszel X^2^ was used to identify the factors associated with deaths from CHD. A value of p<0.05 was considered statistically significant.
4. IBM SPSS Statistics 19 statistical package was used for the statistical analysis [17]. OpenEpi version 3.03 was used to calculate the odds ratio and confidence intervals [18].

## Results

### Trends in mortality from CHD and descriptive analysis

**Fig 1:**
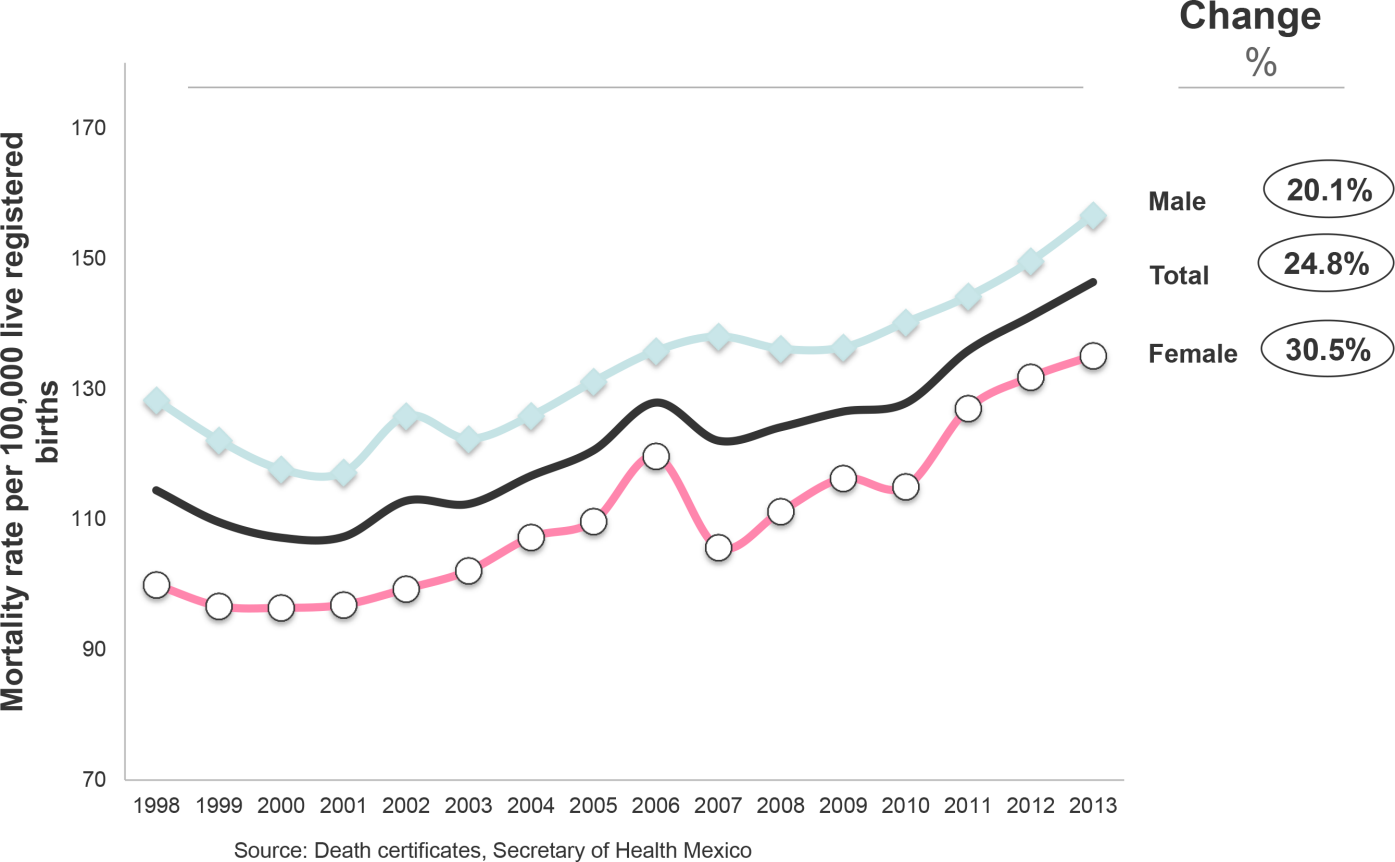
Infant mortality rate from congenital heart disease by sex. Deaths per 100,000 live registered births, Mexico 1998–2013.

A total of 41,717,421 births were registered from 1998 to 2013 and 50,759 deaths from CHD were diagnosed, resulting in a mortality rate for the period of 121.7 per 100,000 LB. Fig 1 shows an increasing trend in the CHD mortality rate over time for children under 1 year of age in Mexico—from 114.4 per 100,000 LB in the year 1998 to 146.4 per 100,000 LB in the year 2013, an increase of 24.8%. Fig 1 also shows a higher overall CHD mortality rate for males than for females. While the mortality rate for both sexes increased from 1998 to 2013, the increase was greater for females.

Table 1 presents the change in the CHD infant mortality rate from 1998 to 2013 in the 32 states in Mexico. Durango and Nayarit registered the highest increases in mortality from CHD (roughly 150 to 180%) while 11 states registered decreases in the CHD mortality rate. In the year 2013, a total of 1,876 deaths from CHD (52%) occurred in Mexico City and five states (State of Mexico, Jalisco, Puebla, Guanajuato and Veracruz).

**Table 1:** Mortality rate from congenital heart disease by state, Mexico 1998 and 2013.

	State	1998		2013		CARG†
		Rate per 100,000 LB*	(n)	Rate per 100,000 LB*	(n)	1998–2013
1	Aguascalientes	138.5	35	106.6	28	-26.0
2	Baja California	141.5	82	132.0	81	-6.9
3	Baja California Sur	110.0	11	104.9	13	-4.8
4	Campeche	90.7	17	195.7	37	78.9
5	Coahuila	88.2	52	104.2	65	16.8
6	Colima	229.9	29	60.5	8	-127.7
7	Chiapas	63.1	84	87.5	143	33.0
8	Chihuahua	90.5	72	131.8	87	38.1
9	Mexico City	162.3	293	188.5	272	15.0
10	Durango	32.7	15	132.4	52	146.5
11	Guanajuato	161.4	216	145.6	172	-10.3
12	Guerrero	39.9	39	100.0	102	94.7
13	Hidalgo	81.0	54	127.8	75	46.3
14	Jalisco	140.7	231	163.9	260	15.3
15	State of Mexico	155.9	517	215.4	681	32.7
16	Michoacán	74.1	91	111.7	117	41.6
17	Morelos	161.8	55	131.8	49	-20.4
18	Nayarit	27.8	7	149.5	36	178.1
19	Nuevo León	143.4	130	137.9	129	-3.9
20	Oaxaca	92.4	98	124.1	114	29.7
21	Puebla	121.6	186	202.1	280	51.7
22	Querétaro	176.2	68	151.7	63	-14.9
23	Quintana Roo	135.3	30	136.3	38	0.7
24	San Luis Potosí	127.2	84	118.0	64	-7.4
25	Sinaloa	46.4	34	115.6	65	94.1
26	Sonora	113.1	63	127.7	67	12.2
27	Tabasco	117.2	58	148.1	77	23.6
28	Tamaulipas	92.9	59	134.0	80	37.1
29	Tlaxcala	125.0	35	122.8	33	-1.8
30	Veracruz	83.5	181	132.2	211	46.7
31	Yucatán	150.8	57	143.5	55	-4.9
32	Zacatecas	93.0	36	111.9	39	18.5
	**National**	**114.4**	**3019**	**146.4**	**3593**	**24.8**

*LB = Live registered births

**†**CARG = Compounded annual rate of growth

Source: INEGI/SSA

**Fig 2:**
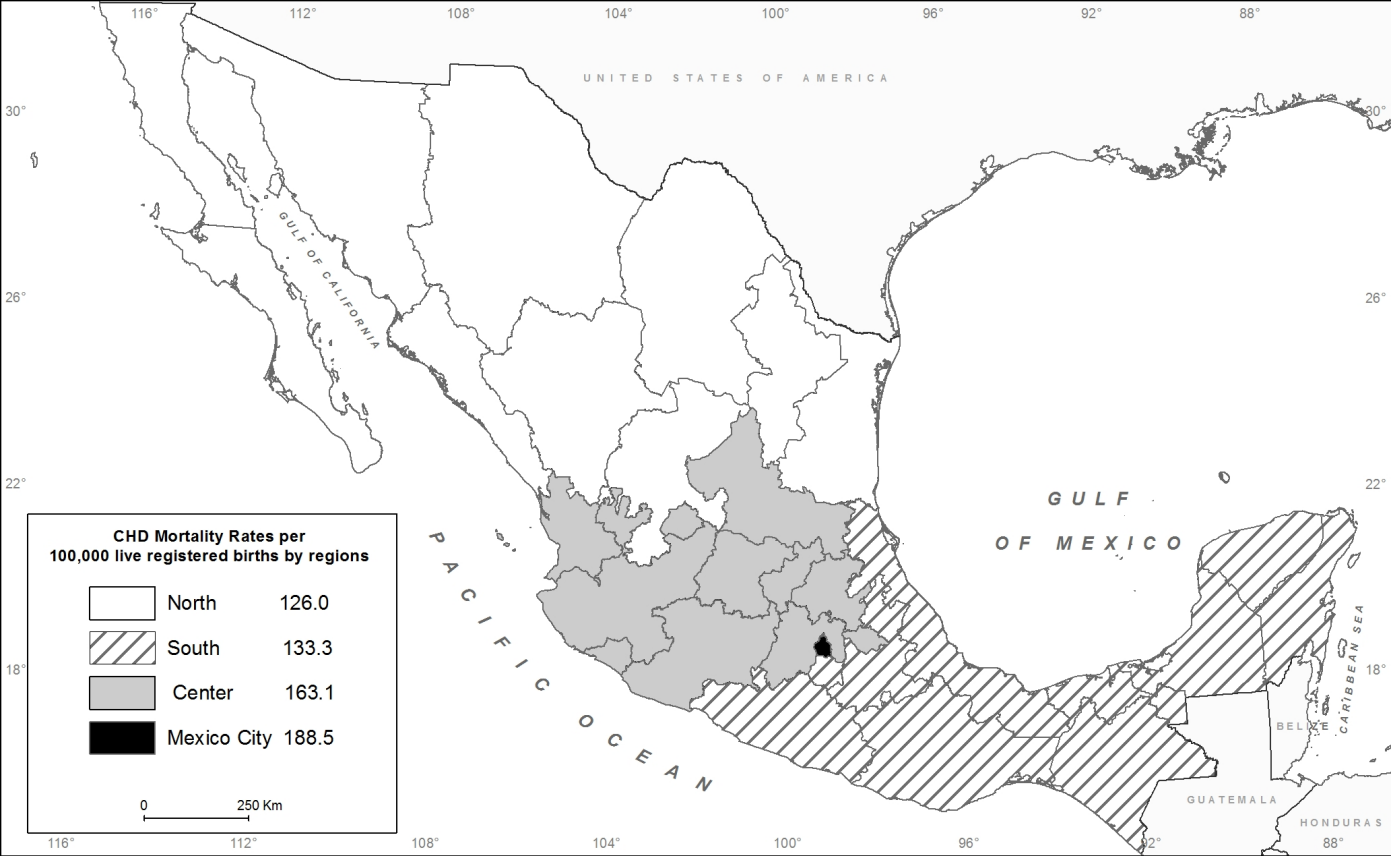
Infant mortality rate from congenital heart disease by region. Deaths per 100,000 live registered births, Mexico, 2013. Reprinted from Instituto de Geografía de la Universidad Nacional Autonóma de México under the Creative Commons Attribution License CC BY 3.0, with permission from Stephan André Couturier, original copyright 2015.

Fig 2 presents the distribution of the CHD mortality rates in the study regions for the year 2013.

Table 2 presents some of the socio-demographic characteristics of infants who died from CHD in 2013. Mexico City reported the highest rate (188.5 per 100,000 LB) and the North reported the lowest (126 per 100,000 LB) for that year.

**Table 2:** Social and demographic characteristics of infant deaths from congenital heart disease. Mexico 2013.

Variable		CHD deaths*(n)	Births (n)	Rate†
**Total**		3,593	2,454,929	146.4
**Sex**				
	Female	1,638	1,212,556	135.1
	Male	1,946	1,242,232	156.7
	NS¶	9	141	-
**Regions**				
	North	678	538,212	126.0
	Central	1,537	942,608	163.1
	Mexico City	272	144,278	188.5
	South	1,106	829,831	133.3
**Area**				
	Urban	2,813	2,047,323	137.4
	Rural	703	218,429	321.8
	NS¶	77	189,177	-
**Birth site**				
	Public hospital	2,508	1,829,008	137.1
	Private hospital	241	317,113	76.0
	Home and others	802	167,104	479.9
	NS¶	42	141,703	

* CHD = Congenital heart disease

**†** Rate per 100,000 live registered births

¶NS = Not-specified

Source: INEGI/SSA

The majority of births as well as deaths from CHD occurred in the urban area. In 2013, there were roughly four times more deaths from CHD in this area than in the rural area. Nevertheless, the mortality rates from CHD were higher in the rural area.

In the year 2013, a total of 69.8% of infant deaths from CHD occurred in public hospitals and 6.7% in private hospitals. The latter had the lowest CHD mortality rate while home births had the highest rate—22.3% of total deaths from CHD. Home and other non-institutional births represented 6.8% of the total births.

In the year 2003, the rate of pediatricians, surgeons and cardiologists per 1,000 LB was 2.4, 1.9 and 0.3, respectively. These rates increased to 4.0, 3.6 and 0.6 by the year 2012, respectively.

### Specific causes of death from CHD

**Fig 3:**
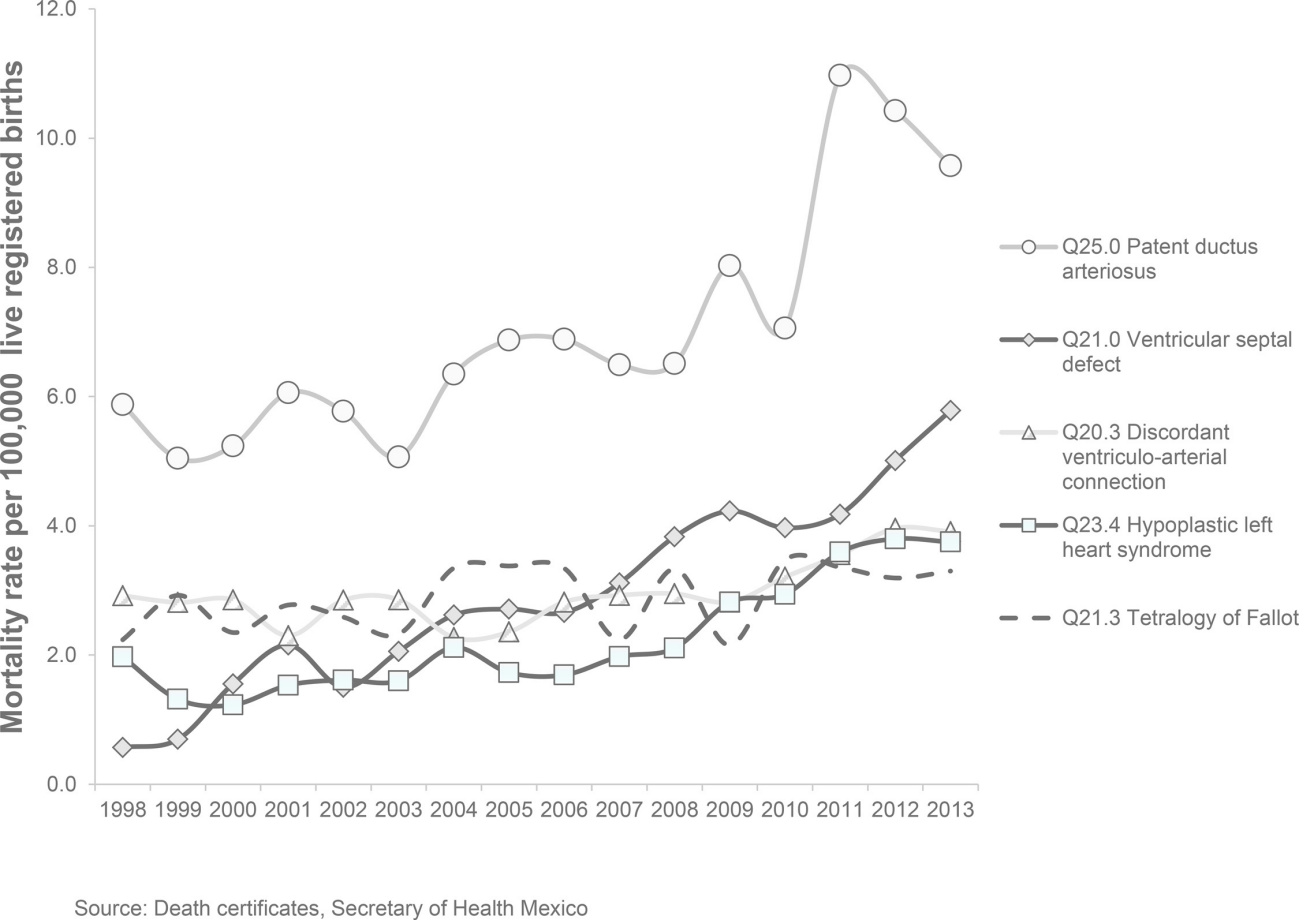
Main causes of infant mortality from congenital heart disease, Mexico 1998–2013.

Fig 3 presents the temporal analysis of specific and individual causes of death from CHD for the period 1998–2013. There was an increase in infant deaths from CHD with left-to-right shunt—specifically, patent ductus arteriosus and ventricular septal defect—which were the main causes of death in 2013, followed by discordant ventriculo-arterial connections and obstruction of the left heart.

According to an analysis of causes of death from CHD for the year 2013, based on the adaptation of the functional classification proposed by Reller et al [15], CHD with left-to-right shunt were the main causes of death in the first year of life. Topping the list were patent ductus arteriosus, ventricular septal defect and auricular septal defect (Table 3). Cyanotic heart diseases, as a group, took second place, most notably discordant ventriculo-arterial connection and Tetralogy of Fallot.

**Table 3:** Functional classification of deaths from congenital heart disease in children under 1 year of age. Rates and frequencies, Mexico 2013.

ICD-	Cause	Male	Female	NS*	Total	Male	Female	Total
10		n	n	n	n	rate†	rate†	rate†
**I**	**LEFT TO RIGHT SHUNT**	**250**	**237**	**0**	**487**	**20.1**	**19.5**	**19.8**
Q25.0	Patent ductus arteriosus	107	128	0	235	8.6	10.6	9.6
Q21.0	Ventricular septal defect	78	64	0	142	6.3	5.3	5.8
Q21.1	Atrial septal defect	38	22	0	60	3.1	1.8	2.4
Q21.2	Atrioventricular septal defect	25	17	0	42	2.0	1.4	1.7
Q21.9	Congenital malformations of cardiac	0	5	0	5	0.0	0.4	0.2
	septum, unspecified							
Q21.4	Aortopulmonary septal defect	1	1	0	2	0.1	0.1	0.1
Q21.8	Other congenital malformations of	1	0	0	1	0.1	0.0	0.0
	cardiac septum							
**II**	**CYANOTIC CONGENITAL**	**239**	**171**	**0**	**410**	**19.2**	**14.1**	**16.7**
	**HEART DEFECTS**							
Q20.3	Discordant ventriculo-arterial	68	28	0	96	5.5	2.3	3.9
	connections							
Q21.3	Tetralogy of Fallot	41	40	0	81	3.3	3.3	3.3
Q20.4	Double inlet ventricle	35	20	0	55	2.8	1.6	2.2
Q26.4	Anomalous pulmonary venous	22	19	0	41	1.8	1.6	1.7
	connection, unspecified							
Q20.0	Common arterial trunk	18	13	0	31	1.4	1.1	1.3
Q26.2	Total anomalous pulmonary venous	17	14	0	31	1.4	1.2	1.3
	connection							
Q22.5	Ebstein anomaly	10	18	0	28	0.8	1.5	1.1
Q22.4	Congenital tricuspid stenosis	16	7	0	23	1.3	0.6	0.9
Q20.9	Congenital malformations of cardiac	5	5	0	10	0.4	0.4	0.4
	chambers and connections,							
	unspecified							
Q20.8	Other congenital malformations of	3	3	0	6	0.2	0.2	0.2
	cardiac chambers and connections							
Q20.5	Discordant auriculoventricular	3	1	0	4	0.2	0.1	0.2
	connection							
Q20.1	Transposition of the great vessels in	1	3	0	4	0.1	0.2	0.2
	the right ventricle							
**III**	**LEFT HEART OBSTRUCTIVE DEFECTS**	**131**	**96**	**0**	**227**	**10.5**	**7.9**	**9.2**
Q23.4	Hypoplastic left heart	58	34	0	92	4.7	2.8	3.7
	syndrome							
Q25.1	Coarctation of aorta	41	38	0	79	3.3	3.1	3.2
Q25.4	Other congenital malformations of	12	10	0	22	1.0	0.8	0.9
	aorta							
Q23.0	Congenital stenosis of aortic valve	10	8	0	18	0.8	0.7	0.7
Q23.2	Congenital mitral stenosis	4	2	0	6	0.3	0.2	0.2
Q23.3	Congenital mitral insufficiency	3	2	0	5	0.2	0.2	0.2
Q23.9	Congenital malformations of aortic	1	2	0	3	0.1	0.2	0.1
	and mitral valves, unspecified							
Q23.1	Congenital insufficiency of aortic	2	0	0	2	0.2	0.0	0.1
	valve							
**IV**	**RIGHT HEART OBSTRUCTIVE**	**60**	**59**	**0**	**119**	**4.8**	**4.9**	**4.8**
	**DEFECTS**							
Q25.5	Atresia of pulmonary artery	32	32	0	64	2.6	2.6	2.6
Q22.8	Other congenital malformations of	10	3	0	13	0.8	0.2	0.5
	tricuspide valves							
Q22.6	Hypoplastic right heart syndrome	6	7	0	13	0.5	0.6	0.5
Q22.0	Pulmonary valve atresia	6	6	0	12	0.5	0.5	0.5
Q25.6	Stenosis of pulmonary artery	4	4	0	8	0.3	0.3	0.3
Q22.1	Congenital pulmonary valve stenosis	1	3	0	4	0.1	0.2	0.2
Q25.7	Other congenital malformations of	1	2	0	3	0.1	0.2	0.1
	pulmonary artery							
Q22.3	Other congenital malformations of pulmonary valve	0	2	0	2	0.0	0.2	0.1
	**NON CLASSIFICABLE**	60	35	0	95	4.8	2.9	3.9
Q24.8	Other congenital heart malformations	51	32	0	83	4.1	2.6	3.4
Q24.6	Congenital heart block	4	0	0	4	0.3	0.0	0.2
Q24.5	Malformation of coronary vessels	3	0	0	3	0.2	0.0	0.1
Q26.9	Congenital malformations of great veins,	2	3	0	5	0.2	0.2	0.2
	unspecified							
Q27	Other congenital malformations of	3	4	0	7	0.2	0.3	0.3
	peripheral vascular system							
Q28	Other congenital malformations of	2	3	0	5	0.2	0.2	0.2
	circulatory system							
Q24.9	Congenital malformations of heart,	1201	1033	9	2243	96.7	85.2	91.4
	unspecified							
	**Total**	**1946**	**1638**	**9**	**3593**	**156.7**	**135.1**	**146.4**

*NS = Not specified

**†**Rate per 100,000 live registered births

Source: INEGI/SSA

### CHD deaths by age at death

Table 4 presents the distribution of the specific causes of death from CHD by age at death for the year 2013. Patent ductus arteriosus, obstructive defects of the left heart, discordant ventriculo-arterial connections represented the majority of the known causes of early neonatal death (under 7 days of life). Patent ductus arteriosus was the main cause of death during the neonatal period as well as during the first year of life. Ventricular septal defect, patent ductus arteriosus and coarctation of the aorta were the main causes during the post-neonatal period. Over 60% of the death certificates did not contain a specific diagnosis of the CHD, and this information was missing on 90% of deaths occurring on the first day of life.

**Table 4:** Main causes of infant mortality from congenital heart disease by age at death (frequencies and proportions). Mexico 2013.

	ICD-10	Cause	Less than 24 hs		1–6 days		7–27 days		28 days-11 months		Less than 1 year	
			n	(%)	n	(%)	n	(%)	n	(%)	N	(%)
1	Q25.0	Patent ductus	4	1.1	45	6.4	93	11.9	93	5.3	235	6.5
		arteriosus										
2	Q21.0	Ventricular septal	1	0.3	9	1.3	27	3.5	105	6.0	142	4.0
		defect										
3	Q20.3	Discordant ventriculo-	3	0.9	19	2.7	30	3.8	44	2.5	96	2.7
		arterial connections										
4	Q23.4	Hypoplastic left heart syndrome	5	1.4	35	5.0	28	3.6	24	1.4	92	2.6
5	Q21.3	Coarctation of aorta	2	0.6	9	1.3	14	1.8	56	3.2	81	2.3
6	Q25.1	Tetralogy of Fallot	1	0.3	15	2.1	31	4.0	32	1.8	79	2.2
7	Q25.5	Atresia of pulmonary artery	3	0.9	12	1.7	17	2.2	32	1.8	64	1.8
8	Q21.1	Atrial septal defect	1	0.3	8	1.1	19	2.4	32	1.8	60	1.7
9	Q20.4	Double inlet ventricle	0	0.0	15	2.1	7	0.9	33	1.9	55	1.5
10	Q21.2	Atrioventricular septal defect	0	0.0	4	0.6	8	1.0	30	1.7	42	1.2
11	Q26.4	Common arterial trunk	0	0.0	5	0.7	7	0.9	29	1.6	41	1.1
12	Q20.0	Anomalous pulmonary,	0	0.0	0	0.0	10	1.3	21	1.2	31	0.9
		venous connection										
		unspecified										
13	Q26.2	Congenital tricuspid	1	0.3	3	0.4	5	0.6	22	1.2	31	0.9
		Stenosis										
14	Q22.5	Ebstein anomaly	2	0.6	6	0.9	10	1.3	10	0.6	28	0.8
15	Q22.4	Congenital stenosis of aortic valve	0	0.0	1	0.1	6	0.8	16	0.9	23	0.6
		**Subtotal**	**23**	**6.6**	**186**	**26.6**	**312**	**39.9**	**579**	**32.9**	**1100**	**30.6**
	Q24.9	Congenital	310	88.3	471	67.5	408	52.2	1054	59.8	2243	62.4
		malformation of heart, unspecified										
		The rest of Q20-Q28	18	5.1	41	5.9	62	7.9	129	7.3	250	7.0
		**Total**	**351**	**100**	**698**	**100**	**782**	**100**	**1762**	**100**	**3593**	**100**

Source: INEGI/SSA

In 2013, approximately 10 children under 1 year of age died from CHD per day, on average. Most of the deaths per day occurred on the day of birth—351 deaths per day, representing approximately 10% of total deaths—followed by the period corresponding to day 1 through 6 of life with an average of 116 deaths per day from CHD, representing 19.4% of total deaths from CHD.

### Factors associated with mortality from CHD

Factors (OR; 95% CI) associated with mortality from CHD were, in order of magnitude: non-institutional birth (6.34;5.49–7.32) as compared to private hospital, rural area (2.35;2.16–2.55) as compared to urban area, birth in public hospital (2.35;2.16–2.55) as compared to private hospital, and male sex (1.16;1.09–1.24) as compared to females.

## Discussion

### Trends in mortality

In 2013 in Mexico, congenital anomalies were responsible for 24% of infant mortality and CHD represented 55% of total deaths from congenital anomalies among children under 1 year of age [10]. From 1998 to 2013, a significant increase (24.8%) in mortality from CHD among children under 1 year of age was detected. And by the year 2013, the CHD mortality rate for children under 1 year of age was 146.4 per 100,000 LB—3.5 times higher than that reported for the U.S. from 1999 to 2006 (41.5 per 100,000 LB) [19]. Two interesting studies that have investigated infant mortality from CHD in Mexico also show that it is on the rise. Nevertheless, one [20] of these only included Mexican municipalities presenting high mortality from congenital anomalies and the other [21] did not include CHD corresponding to International Classification of Diseases (ICD-10) code Q25 (congenital anomalies of great arteries), which constitutes a significant proportion (11.5%) of deaths from CHD among children under 1 year of age. These studies also did not include updated information from the past decade in Mexico, nor did they include a detailed analysis of the specific causes of CHD or the age at death. Thus, the information provided by the work herein is very important because it gives a current and complete national overview of this public health problem.

Also notable is that mortality from CHD increased roughly 150 to 180% in some of the states. This information is important to improve planning related to health services in order to address the demand created by this serious public health problem. Furthermore, it is important to perform epidemiological studies to understand the high risk of CHD observed in certain geographic areas. In 2013, 52% percent of the deaths from CHD occurred in Mexico City and five states. It is of interest that lower-income states (Guerrero, Oaxaca Chiapas and others) had lower CHD mortality rates than states with a higher gross domestic product per capita (Mexico City, Mexico State, Campeche) [9]. This could be related to either the presence of high risk genetic or environmental factors in the latter states or, alternatively, it might represent underreporting of deaths in states with fewer resources. A study by Hernandez et al [22] detected that 22.6% (95% confidence interval (CI):12.3–36.2) of deaths among children under 5 years of age were underreported in Mexican municipalities with a very low human development index.

Although we do not have a clear explanation for the observed trend in CHD mortality, risk factors associated with CHD are on the rise in Mexico. For example, diabetes and substance abuse—both known to be associated with CHD [16, 23–25]—are more prevalent now in women of reproductive age than 10 years ago. [23–25] Also, the number of specialists per capita increased over recent years; thus, it is possible that CHD deaths that previously went undetected are now diagnosed and registered as causes of infant mortality. This deserves further research. Meanwhile, factors that reduce the risk of CHD [26] need to be promoted. These include: perinatal consumption of folic acid; avoidance of smoking, alcohol and drugs by the mother; control of maternal obesity and diabetes; and avoidance of taking medications beginning early in pregnancy (certain types of analgesics and medications proven to be teratogenic).

### Causes of death

The main causes of death from CHD are due to their prevalence and lethality. In Mexico, CHD with left-to-right shunt were the main causes of mortality among children under 1 year of age in 2013. Although CHD with left-to-right shunt are not more severe than others types—such as critical and complex CHD—they are more prevalent [3], which may explain our findings.

Patent ductus arteriosus was the leading cause of mortality from CHD. Although it is known that patent ductus arteriosus could be a physiological manifestation of extreme prematurity, when a death certificate indicates this as a cause of death it is clearly due to a pathological problem that would have received treatment [27]. Therefore, this study included patent ductus arteriosus among the types of CHD responsible for infant mortality, although with the limitation that information about the gestational age of patients with specific CHD was not available for the study population. As has been identified in different populations, the persistence of patent ductus arteriosus is a public health problem. Prevalence studies at the global level and in other countries have reported patent ductus arteriosus to be among the three main causes of congenital heart disease [3]. In a population study performed by Gilboa et al [19] in the U.S., patent ductus arteriosus was the fourth cause of infant mortality from CHD (rate of 1.3/100,000 LB) between 1999 and 2006. This indicates the importance of patent ductus arteriosus during the first year of life even in developed countries. In a report on Mexico by Cervantes-Salazar et al [28], patent ductus arteriosus and ventricular septal defects were the CHD that most frequently required a surgical intervention, according to the Mexican Registry of Pediatric Heart Surgery.

Ventricular septal defects became an increasingly more important cause of mortality from CHD over time, representing the second highest rate (5.8/100,000 per LB) by the year 2013. Given its mortality rate of 1.1/100,000 LB in the U.S. [19], this problem should also be addressed globally, since mortality should not be high when detected early and managed appropriately.

Cyanotic CHD, such as discordant ventriculo-arterial connections, and obstructive defects of the left heart, such as left ventricular hypoplasia and coarctation of the aorta, were among the six main causes of mortality from CHD. The latter are complex CHD which require specialized care and could be associated with worse outcomes. This is especially true for left ventricular hypoplasia, which is the main cause of mortality from CHD among children under 1 year of age in the U.S., with a mortality rate of 8.6/100,000 LB [19].

### Age at death

In the year 2013, a total of 3,593 deaths from CHD occurred in Mexico, approximately 10% of which occurred on the first day of life. The analysis of the causes of death during the first 24 hours found that roughly 90% did not have a specific diagnosis, which is a cause for concern. For those that did have a diagnosis, left ventricular hypoplasia, patent ductus arteriosus, discordant ventriculo-arterial connection and pulmonary valve atresia were the leading causes. The lack of a specific diagnosis is a significant weakness in our hospital information system and, therefore, prenatal and early postnatal diagnosis in the first hours of life should be encouraged. This should include a complete physical exam and universal screening for CHD using pulse oximetry [29], a non-invasive procedure which is increasingly used for the timely diagnosis of CHD. Major CHD can be diagnosed during the prenatal period, which provides the opportunity to adequately plan postnatal treatment for these patients. In a systematic review of the literature performed by Holland et al [30], newborns with a prenatal diagnosis of critical congenital heart disease were significantly less likely to die prior to planned cardiac surgery than were those with a comparable postnatal diagnosis (OR, 0.26; 95% CI: 0.08–0.84). Also, a recent paper published by Eckersley et al [31] about a population study in New Zealand showed that early (antenatal or pre-discharge) diagnosis of critical CHD had better outcomes than late diagnosis (post-discharge). This study reported a higher mortality for late diagnosis than for early diagnosis (27% vs 16%, respectively; p<0.04).

Patent ductus arteriosus was the main cause of death from CHD during the neonatal period. The main causes of CHD during the post-neonatal period were ventricular septal defects, patent ductus arteriosus and coarctation of the aorta. A specific diagnosis of the cause of death during these periods was lacking in more than half of the cases. This may indicate a failure to adequately fill out the death certificate or a diagnostic failure by the echocardiography. These problems need to be improved in order to understand the epidemiology of mortality from CHD in Mexico. The absence of this data has also been reported in developed countries. For instance, in the U.S. a specific diagnosis was found to be lacking in as many as 34% of the CHD [19].

The number of deaths from CHD per day reported for different ages indicates the importance of the first week of life as a critical period, when the majority of deaths from CHD occurred, and particularly the first day of life. This situation should enable specialized health teams to design and plan strategies to determine diagnoses during prenatal and early neonatal periods and to provide effective and timely care [32]. To improve the life expectancy of patients with CHD, modern intensive care units are needed as well as health personnel trained to manage them. Finally, the implementation of a good hospital referral system is important.

### Factors associated with mortality

Factors associated with mortality from CHD were identified which indicate the social determinants of health that need to be addressed to improve infant survival. While 6.8% of births occurred at home or at other non-institutional sites, roughly 25% of the children under 1 year of age who died from a CHD in 2013 were born in this setting (data not shown), and the odds of dying from CHD were lower for births at private hospitals than for those at home and in public institutions. This fact leads us to emphasize the importance of implementing the WHO recommendation [33] to examine babies born at home as soon as possible during the first 24 hours of life, and to have three more check-ups thereafter. It is also important to promote institutionalized births and to understand the gap between outcomes at public and private institutions. Better outcomes at private institutions could be explained by better socioeconomic conditions and a better health status of the population using private centers, or these hospitals may use better practices to diagnose and treat CHD. In addition, living in a rural area was associated with 2.35 times more likelihood of dying from CHD compared to children living in urban areas. Meanwhile, a different result was observed in China, where a higher probability of dying (OR 1.20; 95%CI: 1.04–1.05) was associated with urban areas for children under 5 years of age [8]. These differences demonstrate the importance of conducting regional analyses. And finally, being a male—a non-modifiable biological risk factor—was associated with higher mortality, as has been previously reported [7].

National strategies need to focus on these vulnerable populations in the country and provide more investment and infrastructure for public hospitals, as well as eliminate the social barriers to accessing health services. Studies should also be conducted to determine whether rural areas present more exposure to factors that can increase the risk of CHD.

The strengths of this study include its population-based design and covering a long period of time, as well as the use of the ICD-10 on death certificates in Mexico which enabled making international comparisons. Another strength is the functional sub-classification presented. The inclusion of the age at death from CHD and the geographic analysis of these deaths are also very important since they contribute to establishing public health strategies to improve the prognosis of patients with CHD.

The weaknesses of the study include a lack of connection between birth certificates and death certificates in Mexico, not knowing the gestational age of CHD-specific deaths and not having identified deaths from CHD associated with chromosomal abnormalities. Another weakness is the high proportion of unspecified diagnoses, particularly on the first day of life, and the lack of fetal data which certainly underestimates the magnitude of this problem.

## Conclusions

In Mexico, CHD presents a serious public health problem and has been increasing over recent years, as is occurring in several developing countries. Research is needed to understand this finding. The persistence of ductus arteriosus and other CHD with left-to-right shunt are the main causes of death among children under 1 year of age who have CHD. The neonatal period is the stage when most infant deaths from CHD occur.

Improving the early detection and epidemiological monitoring of CHD is also needed in Mexico. And linking the health information system to data from death and birth certificates is indispensable.

Finally, it is important for Mexico’s health system to promote comprehensive postnatal medical visits as recommended by the WHO.
